# Inhibition of Catechol-*O*-methyltransferase Does Not Alter Effort-Related Choice Behavior in a Fixed Ratio/Concurrent Chow Task in Male Mice

**DOI:** 10.3389/fnbeh.2020.00073

**Published:** 2020-05-21

**Authors:** Adrienne C. DeBrosse, Abigail M. Wheeler, James C. Barrow, Gregory V. Carr

**Affiliations:** ^1^Lieber Institute for Brain Development, Johns Hopkins University Medical Campus, Baltimore, MD, United States; ^2^Department of Pharmacology and Molecular Sciences, Johns Hopkins School of Medicine, Baltimore, MD, United States

**Keywords:** dopamine, tolcapone, catechol-*O*-methyltransferase, motivation, touchscreen, prefrontal cortex

## Abstract

Effort-related choice (ERC) tasks allow animals to choose between high-value reinforcers that require high effort to obtain and low-value/low-effort reinforcers. Dopaminergic neuromodulation regulates ERC behavior. The enzyme catechol-*O*-methyltransferase (COMT) metabolizes synaptically-released dopamine. COMT is the predominant regulator of dopamine turnover in regions of the brain with low levels of dopamine transporters (DATs), including the prefrontal cortex (PFC). Here, we evaluated the effects of the COMT inhibitor tolcapone on ERC performance in a touchscreen-based fixed-ratio/concurrent chow task in male mice. In this task, mice were given the choice between engaging in a fixed number of instrumental responses to acquire a strawberry milk reward and consuming standard lab chow concurrently available on the chamber floor. We found no significant effects of tolcapone treatment on either strawberry milk earned or chow consumed compared to vehicle treatment. In contrast, we found that haloperidol decreased instrumental responding for strawberry milk and increased chow consumption as seen in previously published studies. These data suggest that COMT inhibition does not significantly affect effort-related decision making in a fixed-ratio/concurrent chow task in male mice.

## Introduction

Humans and other mammals interact with complex stimuli in their environments and make decisions to maximize economic value and improve survival odds. Economic value is defined as the benefit provided by a good or service relative to the amount of currency required to procure it (Samuelson, 1947). A common type of decision is one where a high-value item and a low-value item of similar quality are both available. The choice is easy when the amount of effort (currency) required to procure both items is the same. Conversely, when the high-value option requires more work to attain, the calculation becomes more complicated because effort-based discounting of the high-value option determines the choice (Walton et al., 2006; Botvinick et al., 2009).

The capacity to make effort-based decisions is disrupted in many psychiatric and neurological disorders, including depression and schizophrenia (Treadway et al., 2012; Fervaha et al., 2013; Gold et al., 2013; Barch et al., 2014). In the case of schizophrenia, the cognitive effort allocated to gaining a reward drops off more steeply than in healthy individuals and is correlated with overall negative symptomatology (Culbreth et al., 2016). These data suggest that an increased understanding of how effort-based decisions are made would potentially improve treatment options for psychiatric disorders.

There are several paradigms for assessing effort-based decision-making in humans and animal models, including the human Effort Expenditure for Rewards Task (EEfRT), rodent T-maze with barriers, and rodent operant concurrent choice tasks (Cousins et al., 1994; Botvinick et al., 2009; Mott et al., 2009; Treadway et al., 2012; Enomoto et al., 2018). Recently, rodent touchscreen-based versions of operant concurrent choice effort-related choice (ERC) tasks have been developed (Heath et al., 2015; Yang et al., 2020).

The dopaminergic system has long been recognized as a critical regulator of ERC performance from studies that utilized systemic manipulations. The D2 antagonist haloperidol biases behavior toward the low-effort low-value option across multiple versions of ERC tasks (Salamone et al., 1991, 1994; Randall et al., 2012; Yang et al., 2020). Similarly, the D1 receptor antagonist ecopipam decreases the choice of the high-effort arm of a T-maze and decreases lever pressing in a fixed ratio (FR)/concurrent chow paradigm (Yohn et al., 2015a). Modulation of dopamine transporter (DAT) activity also affects ERC performance. DAT knockdown mice demonstrate an increased preference for the high-effort high-value reward in an FR/concurrent chow task (Cagniard et al., 2006). Pharmacological treatment with DAT inhibitors also biases rats toward the high-effort reward in a progressive ratio (PR)/concurrent chow task (Sommer et al., 2014).

Experiments targeting the nucleus accumbens (NAc) have demonstrated a particularly strong involvement of the mesolimbic dopaminergic system in ERC behavior. Dopamine depletion in the NAc biases responding toward the low-effort low-reward option (Salamone et al., 1994; Cousins et al., 1996). Local injections of both the D1 antagonist SCH 23390 and the D2 antagonist raclopride into the NAc shell and core decrease lever pressing in rats during an FR/concurrent chow task (Nowend et al., 2001). Direct injections of haloperidol into the NAc likewise decrease lever pressing on an FR5/concurrent chow task (Salamone et al., 1991).

The relationship between NAc dopamine and ERC performance is bidirectional as increased dopaminergic signaling biases responding toward the high-effort high-reward options. Overexpression of the D2 receptor in the NAc increases lever pressing on an ERC task (Trifilieff et al., 2013). Individual rats that respond more highly at baseline during a PR/concurrent chow lever-pressing task show higher levels of D1 receptor activation, measured as changes in DARPP-32 phosphorylation, in the NAc core (Randall et al., 2012). Moreover, in human fMRI studies, the NAc was activated during an effort discounting task in direct relation to the amount of reward that was cued (Botvinick et al., 2009).

The role of DA in the prefrontal cortex (PFC) in ERC performance is less clear. Rodent lesion and human fMRI studies implicate the anterior cingulate cortex (ACC) in regulating ERC performance. Specifically, ACC lesions bias responding toward the low-effort low-value options (Walton et al., 2002, 2003) and the ACC is activated in fMRI studies during effort-based decision-making (Botvinick et al., 2009; Klein-Flügge et al., 2016). Local infusion of the D1 antagonist SCH23390 into the ACC reduces the preference for the high-effort high-value reward in rats (Schweimer and Hauber, 2006). However, the effects of catecholamine depletion (6-hydroxydopamine lesions) in the ACC are ambiguous as one study found that depletion reduces the preference for the high-effort high-value option (Schweimer et al., 2005) while a separate study with more targeted DA depletion, by protecting noradrenergic terminals with desipramine pretreatment, showed no effect of the lesions (Walton et al., 2005).

To our knowledge, there have not been any studies investigating the effects of augmenting PFC dopaminergic activity on ERC performance. The enzyme catechol-*O*-methyltransferase (COMT) is an important regulator of cortical dopaminergic function due to its role in the degradation of synaptically-released dopamine (Männistö and Kaakkola, 1999). Acute inhibition of COMT leads to a decrease in dopamine turnover in regions of the brain with low DAT expression, including the PFC (Yavich et al., 2007; Käenmäki et al., 2010). COMT also plays a significant role in the intertemporal choice and delay discounting (Boettiger et al., 2007; Kayser et al., 2012), but it has not been investigated in an ERC task as monotherapy in humans or animal models.

Touchscreen-based rodent tasks have become staples of behavioral neuroscience (Bussey et al., 1997; Brigman et al., 2008; Hvoslef-Eide et al., 2015). Assays of reward-seeking and motivation have been developed alongside tasks that measure other cognitive processes (Heath et al., 2016; Yang et al., 2020). Our group has begun to use touchscreen-based tasks extensively in drug discovery efforts and drug-induced changes in reward-seeking and motivation are important to consider when interpreting behavioral changes in tasks designed to assay other cognitive domains. Here, we tested the effects of the COMT inhibitor tolcapone in a touchscreen-based ERC task.

## Materials and Methods

### Mice

Eight-week-old male C57BL/6J mice (The Jackson Laboratory, Bar Harbor, ME, USA) were used in all experiments. The mice were housed in disposable polycarbonate caging (Innovive, San Diego, CA, USA) in groups of four. The mice were maintained on a 12/12 light/dark cycle (lights on at 06:00 h). Water was available in the home cage *ad libitum* throughout all experiments. The mice were fed Teklad Irradiated Global 16% Protein Rodent Diet (#2916; Envigo, Indianapolis, IN, USA) in the home cage *ad libitum* until the start of the food restriction protocol. Two separate cohorts of mice (Cohort A, *n* = 8; Cohort B, *n* = 8) were tested in these experiments and all testing was done during the light phase (12:00-16:00 h). All experiments and procedures were approved by the SoBran, Inc., Fairfax, VA, USA Rangos Animal Care and Use Committee and following the *Guide for the Care and Use of Laboratory Animals*.

### Drugs

Tolcapone was synthesized in-house. It was suspended in a vehicle (0.1% Tween^®^ 80, 0.1% XIAMETER™ AFE-1510 silicone antifoam, 1% methylcellulose in water). We dosed tolcapone at 3, 10, and 30 mg/kg. The 30 mg/kg dose has been shown to modulate behavior in both mice and rats in multiple assays (Tunbridge et al., 2004; Lapish et al., 2009; Detrait et al., 2016). Additionally, the exposure levels at 30 mg/kg dose produce over 90% inhibition of COMT activity in rats *in vivo* (Napolitano et al., 2003). Haloperidol was purchased from Sigma–Aldrich (St.Louis, MO, USA). It was dissolved in 1% DMSO in a citrate phosphate buffer (pH 5). Both drugs were administered by intraperitoneal (ip) injection 1 h before the start of behavioral testing. Administration volumes were 10 ml/kg.

### Food Restriction Protocol

Upon arrival at the animal facility, mice were given at least 72 h to acclimate to the colony room before handling by experimenters. Mice were handled and weighed daily from that point forward. After at least 2 days of handling, mice were food restricted to 3 g of chow per mouse per day to maintain 85–90% of their predicted free-feeding weight based on average growth curve data for the strain (The Jackson Laboratory). The daily allowance was in addition to the amount each mouse consumed during ERC sessions. Mice were removed from the standard group housing setup due to aggression or significant deviations from the expected body weight during food restriction. In Cohort A, we split one mouse that was under 85% of the predicted average body weight and two mice that were >90% predicted average body weight while their cage mates were in range. In Cohort B, two mice were split and singly housed due to aggression. To familiarize the mice with the Nesquik^®^ strawberry milk (Nestlé, Vevey, Switzerland) reward used in the ERC task, we introduced the milk to the home cage on 4 × 4 inch weighing paper (VWR, Radnor, PA, USA). The weighing paper was left in the cage until all mice had sampled the strawberry milk. This procedure was repeated for a total of 2 days.

### Operant Chamber Habituation and Initial Touch Training

We adapted the ERC training and task parameters (Table 1) described by Heath et al. (2015). Our training and test schedules were programmed in the Animal Behavior Environment Test System (ABET II; Lafayette Instrument, Lafayette, IN, USA) and run in four Bussey-Saksida Mouse Touch Screen Chambers (Model 80614E, Lafayette Instrument, Lafayette, IN, USA). All training and testing were conducted 5 days per week (Monday–Friday). Mice were habituated to the chambers during two consecutive 20-min sessions in which they were presented with 200 μl of strawberry milk in the reward tray. Mice passed the Habituation stage when they had consumed all of the milk in the tray. After reaching criterion, mice advanced to the Initial Touch stage. During Initial Touch training, mice began to learn the cues of a trial, including a lit tray light when strawberry milk was dispensed into the tray after the expiration of a 30-s trial timer. Mice could earn more reward by making a nose poke response at the center square in a 5-mask array on the touchscreen (30 μl of strawberry milk for touching while omissions resulted in 10 μl of the reward). For Initial Touch training, a passing criterion was set at 30 completed trials within the 60-min session.

**Table 1:** Order of training and testing phases.

Phase	Session duration	Criterion
Handling & food restriction	-	Mouse samples the strawberry milk on weight paper
Operant chamber habituation	20 min	Mouse eats all 200 μl strawberry milk from the tray
Initial touch training	60 min	Mouse advances through 30 trials
Fixed ratio training	30 min	Mouse completes one 30 trial session of the FR1, FR2, FR3 each and three consecutive 30 trial sessions of the FR5 schedule
Baseline ERC (FR1, FR2, FR4, FR8, FR16)	30 min	Two sessions on each reinforcement schedule (Cohort A only)
ERC with drug treatment (FR4)	30 min	No passing criterion, fixed schedules

*Cohort A completed all phases in order while Cohort B did not do the Baseline ERC phase*.

### Fixed Ratio Training

During FR training, mice were required to nose poke the illuminated center square of the 5-mask array to receive a reward (10 μl). Each session had an assigned response requirement or number of touches to the center square that was needed to earn each reward. For example, during an FR2 schedule, a mouse would receive one reward after two responses at the center square. Response requirements remained constant within a session and increased as mice passed through training. We trained the mice on response requirements of one (FR1), two (FR2), three (FR3), and five (FR5) responses. The criterion was set at 30 completed trials within a 30-min session. Mice were required to reach criterion on one FR1, one FR2, one FR3, and three consecutive FR5 sessions to advance on to the ERC task. In cases where mice reached the FR5 criterion on days other than Friday, we maintained mice on the FR5 schedule until Monday of the following week to avoid confounding schedule and day of the week in the next phase of the experiment.

### The Effort-Related Choice Task

The ERC task utilized FR schedules with the addition of a preweighed ~3 g pellet of standard laboratory chow available on the floor of the touchscreen chamber. Before the mouse was put into the chamber, the chow was placed between the front and back infrared beams. To counterbalance starting chow position across chambers, chow was placed next to either the back or front wall on the right or left side of the chamber. Mice had the choice of responding at the touchscreen to earn strawberry milk rewards based on the specific FR schedule or consume the freely available chow. Each session was 30 min in duration. After each session, chow crumbs were swept down into the catch tray beneath the chamber and weighed together with the remaining chow to determine how much was consumed during the session. We continued to feed mice 3 g chow/mouse in the home cage following the experiment, as well, to keep them in the 85–90% predicted free-feeding body weight range.

The mice in these experiments were split into two cohorts (A and B) that were tested on separate protocols to answer specific experimental questions.

#### Cohort A

During the first phase of ERC testing, the mice were tested on FR schedules with increasing response requirements over 5 days (FR1, FR2, FR4, FR8, and FR16). The same schedules were presented during the next week but in reverse order (FR16, FR8, FR4, FR2, and FR1). Next, we tested the effect of tolcapone on choice at three doses (3, 10, and 30 mg/kg) using the FR4 schedule over the following 3 weeks. We interleaved drug/vehicle dosing days (Tuesday/Thursday) with no-injection baseline days (Monday/Wednesday/Friday). We also tested haloperidol (0.1 mg/kg) as a comparator to validate our protocol.

#### Cohort B

This cohort proceeded directly from training to the drug treatment phase. The drug treatment phase was identical to Cohort A.

### Statistical Analysis

All statistical analyses were conducted in GraphPad Prism 8 (GraphPad Software, San Diego, CA, USA). We used *t*-tests to analyze the habituation and training data. Welch’s *t-tests* were used for the Habituation, Initial Operant, FR2, and FR3 stages due to unequal variances between the groups. The baseline ERC and ERC-tolcapone data were analyzed using repeated measures one-way ANOVAs with Geisser-Greenhouse corrections. Tukey’s tests were used for *post hoc* analyses. We utilized paired *t*-tests to analyze the haloperidol data. In analyses where performance following drug or vehicle treatment was compared to baseline performance, the baseline was calculated by averaging the performance from the three baseline testing days during the same week as the drug/vehicle session. During the drug treatment phase, we found no differences in performance between Cohort A and Cohort B, so we combined them for the statistical analyses of the effects of tolcapone and haloperidol. One mouse was removed from the analysis of the tolcapone experiment because it did not receive the 30 mg/kg dose due to experimenter error. The statistical significance threshold was set at *p* < 0.05. Data are presented as the mean ± SEM.

## Results

### Training

All mice progressed through training and advanced to the ERC task. Data for the training stages are shown in Table 2. There was no difference in the number of sessions to reach criterion during the Habituation stage (*t*_(7)_ = 1.871, *p* = 0.1036), however, Cohort B required more total sessions to pass the Initial Operant (*t*_(7)_ = 3.416, *p* = 0.0112) and the FR1 (*t*_(14)_ = 3.326, *p* = 0.0050) stages. These group differences did not persist beyond these early training stages as there were no differences in the sessions required to reach criterion in the FR2 (*t*_(7)_ = 1.000, *p* = 0.3506), FR3 (*t*_(7)_ = 2.049, *p* = 0.0796), or FR5 (*t*_(14)_ = 0.8215, *p* = 0.4251) stages.

**Table 2:** Days to criterion.

Cohort	Habituation	Initial operant	FR1	FR2	FR3	FR5
A	2.0 ± 0	1.0 ± 0	2.2 ± 0.25	1.2 ± 0.25	1.0 ± 0	5.5 ± 0.78
B	2.5 ± 0.27	1.6 ± 0.18*	4.0 ± 0.46**	1.0 ± 0	1.8 ± 0.37	6.9 ± 1.5

*p < 0.05 and **p < 0.01 compared to Cohort A. n = 8/cohort.

### Baseline ERC Performance

**Figure 1:**
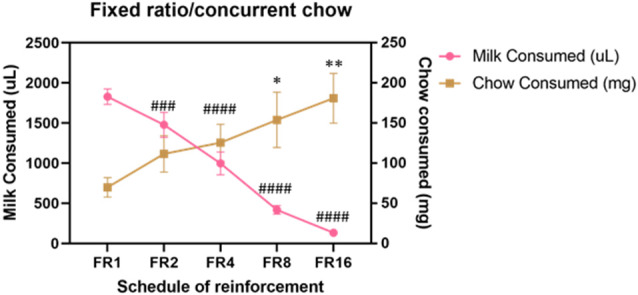
Baseline performance on variable fixed ratio (FR)/concurrent chow sessions. Strawberry milk consumed is plotted on the left axis and chow consumed is plotted on the right axis. Each mouse was tested twice at each FR requirement. The two sessions were averaged to produce a single value for each mouse. Strawberry milk consumption under each FR requirement was significantly different from all of the others. All group comparisons for strawberry milk consumption were *p* < 0.0001 compared to FR1 except for FR1 vs. FR2 (*p* = 0.0087). Chow consumption increased as the FR requirement for strawberry milk increased. Chow consumption in the FR8 (*p* = 0.0465) and FR16 (*p* = 0.0047) sessions were significantly different then the FR1 session. ^###^*p* < 0.001 and ^####^*p* < 0.0001 compared to strawberry milk consumption on during FR1 sessions. *p < 0.05 and **p < 0.01 compared to chow consumption during FR1 sessions. *N* = 8.

Cohort A was first tested on an ERC task where the ratio requirement to earn the strawberry milk reward was varied across sessions. Mice had two sessions each with five different FR response requirements (FR1, FR2, FR4, FR8, and FR16) that we presented in ascending order (first week) and descending order (second week). There were no differences in strawberry milk or chow consumption between the first session (ascending) and the second session (descending) for any of the response requirements (Table 3). For follow-up analyses, we averaged the two sessions on each response requirement for each mouse. As expected, the number of rewards earned, expressed as strawberry milk consumed, decreased as the ratio requirement increased (*F*_(4,28)_ = 108.7, *p* < 0.0001). The mean value of milk consumed at each ratio requirement was significantly different from all of the other ratios tested (Figure 1). Also, as the work requirement for the strawberry milk increased, consumption of the freely available chow increased (*F*_(4,28)_ = 4.380, *p* = 0.0071; Figure 1). *Post hoc* comparisons showed that the amount of chow consumed during FR1 sessions was significantly lower than the amount consumed on FR8 (*p* = 0.0465) and FR16 (*p* = 0.0047) sessions.

**Table 3:** Food consumption during ERC curve setting.

	Consumption				Paired *t*-test results for Session 2−Session 1 (*df* = 7)			
	Session 1		Session 2		Milk		Chow	
Response ratio	Milk (μl)	Chow (mg)	Milk (μl)	Chow (mg)	*t*	*p*-value	*t*	*p*-value
FR1	1,740 ± 97.98	66.68 ± 13.54	1,915 ± 148.2	73.23 ± 15.09	1.072	0.3193	0.4254	0.6833
FR2	1,493 ± 153.9	60.19 ± 9.631	1,463 ± 193.4	162.9 ± 46.34	0.1851	0.8584	2.086	0.0754
FR4	972.5 ± 143.6	85.99 ± 17.40	1,023 ± 148.4	164.9 ± 40.81	0.7372	0.485	1.844	0.1077
FR8	448.0 ± 65.22	131.6 ± 34.81	392.5 ± 53.44	176.2 ± 44.62	0.9751	0.3620	1.093	0.3106
FR16	142.5 ± 14.36	183.7 ± 39.83	122.5 ± 17.90	178.1 ± 39.31	0.8584	0.4191	0.1128	0.9134

### Effects of Tolcapone on ERC Performance

For the drug treatment stage of testing, we chose to use the FR4 schedule for all sessions. Based on the data collected in the first experiment, the FR4 schedule was predicted to produce a baseline level of performance that would allow us to measure significant increases or decreases in both milk and chow consumption.

**Figure 2:**
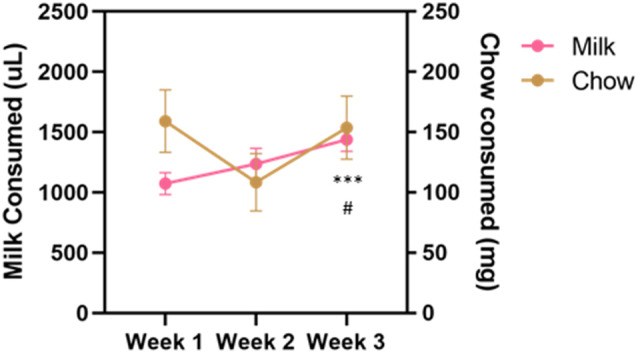
Average consumption during baseline sessions during the drug treatment stage. Strawberry milk consumed is plotted on the left axis and chow consumed is plotted on the right axis. Each mouse had three baseline sessions (Monday, Wednesday, Friday) each week. Consumption of both strawberry milk and chow was averaged across the three sessions within a week. Baseline strawberry milk consumption increased over the testing period with a significant difference between Week 3 and Week 1 (*p* = 0.0009) and Week 3 and Week 2 (*p* = 0.0255). ^#^*p* < 0.05 compared to Week 2 and ***p < 0.001 compared to Week 1. *N* = 15.

Vehicle, tolcapone (3, 10, 30 mg/kg), and haloperidol (0.1 mg/kg) were administered over the course of 3 weeks. We conducted three baseline (no drug or vehicle) ERC sessions each week. We found that the weekly average of strawberry milk consumption increased over the 3 weeks (*F*_(1.964,27.49)_ = 12.49, *p* < 0.0001; Figure 2). In contrast, chow consumption did not change over time (*F*_(1.413,19.78)_ = 2.253, *p* = 0.1427; Figure 2). To determine if there were fluctuations in baseline consumption from session-to-session, we also compared across the three sessions within each week and these data are presented in Table 4. We found there were no differences within any of the weeks on strawberry milk consumption (Week 1: *F*_(1.722,25.83)_ = 0.3119, *p* = 0.7025; Week 2: *F*_(1.393,20.89)_ = 1.976, *p* = 0.1720; Week 3: *F*_(1.746,26.18)_ = 2.747, *p* = 0.0889) or chow consumption (Week 1: *F*_(1.738,26.07)_ = 0.8410, *p* = 0.4279; Week 2: *F*_(1.653,24.79)_ = 1.872, *p* = 0.1795; Week 3: *F*_(1.362,20.42)_ = 1.002, *p* = 0.3549). Therefore, we decided to also analyze the data by normalizing each vehicle or drug treatment session to the average of the three baseline sessions during the same week.

**Table 4:** Baseline sessions.

	Week 1			Week 2			Week 3		
Food Type	Session 1	Session 2	Session 3	Session 1	Session 2	Session 3	Session 1	Session 2	Session 3
Milk (μl)	1,080 ± 102.1	1,107 ± 122.2	1,037 ± 113.1	1,224 ± 113.8	1,116 ± 144.2	1,312 ± 190.8	1,457 ± 144.5	1,436 ± 126.3	1,277 ± 111.0
Chow (mg)	181.0 ± 34.89	157.5 ± 28.88	151.4 ± 26.94	136.8 ± 37.06	96.20 ± 27.19	82.52 ± 17.25	124.1 ± 33.70	194.4 ± 44.24	137.9 ± 28.43

**Figure 3:**
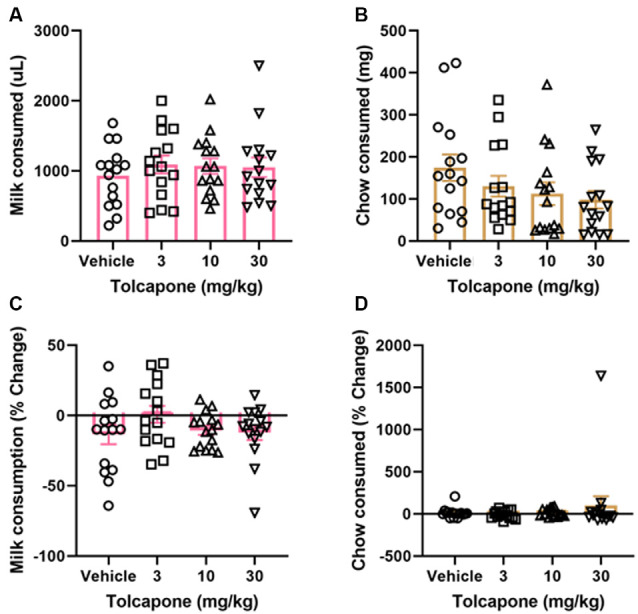
Effects of tolcapone on performance in FR4/concurrent chow sessions. Tolcapone (3, 10, and 30 mg/kg) did not affect strawberry milk **(A)** or chow consumed **(B)**. We also analyzed the data by normalizing performance to baseline (no vehicle or drug injection) sessions. After normalization, there was still no effect of tolcapone on strawberry milk **(C)** or chow consumed **(D)**. *N* = 15.

Tolcapone did not alter the amount of strawberry milk earned (*F*_(1.838,25.74)_ = 1.153, *p* = 0.3272; Figure 3A) or chow consumed (*F*_(2.294,32.12)_ = 2.331, *p* = 0.1068) at any dose tested (3, 10, 30 mg/kg; Figure 3B). Normalizing to the baseline sessions did not change the results of the comparison. There were still no significant changes in strawberry milk earned (*F*_(2.247,31.45)_ = 1.621, *p* = 0.2118; Figure 3C) or chow consumed (*F*_(1.044,14.62)_ = 0.7226, *p* = 0.4149; Figure 3D).

### Effects of Haloperidol on ERC Performance

**Figure 4:**
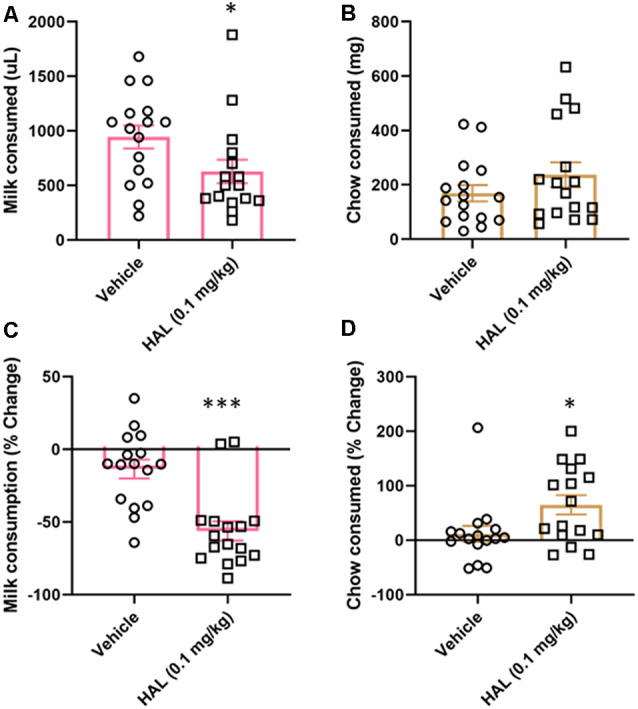
Effects of haloperidol on performance in FR4/concurrent chow sessions. Haloperidol (0.1 mg/kg) decreased strawberry milk consumption **(A)** and did not significantly change chow consumption **(B)**. We also analyzed the data by normalizing performance to baseline (no vehicle or drug injection) sessions. After normalization, there was still a significant decrease in strawberry milk consumption **(C)** and also an increase in chow consumed **(D)**. *p < 0.05; ***p = 0.001 compared to vehicle. *N* = 16.

To validate our ERC protocol, we also tested the effects of haloperidol (0.1 mg/kg) as a positive control. Haloperidol has been shown to decrease responding for the high-value option in ERC tasks, including a recent study using a touchscreen-based task (Yang et al., 2020). Here, we found that haloperidol decreased the amount of strawberry milk earned (*t*_(15)_ = 2.477, *p* = 0.0272; Figure 4A), but did not significantly increase the amount of chow consumed (*t*_(15)_ = 1.685, *p* = 0.1126; Figure 4B).

As with tolcapone, we also compared the effects of haloperidol on vehicle treatment normalized to baseline consumption. We found that haloperidol decreased the amount of strawberry milk earned (*t*_(15)_ = 4.657, *p* = 0.0003; Figure 4C) and it also significantly increased chow consumption (*t*_(15)_ = 2.221, *p* = 0.0422; Figure 4D).

## Discussion

Here, we describe a series of experiments using a modified touchscreen-based ERC task. In agreement with previous studies using multiple ERC tasks, we found that male mice modulate their consumption of a highly palatable (high value) food source based on the amount of work required to attain the food. As the work requirement for the high-value food increased, mice both decreased their consumption of the high-value food and increased the consumption of freely available standard chow. We validated our novel ERC task by using haloperidol as a positive control. We found that haloperidol decreased the amount of high-value food earned and consumed and increased the amount of chow consumed. These effects of haloperidol have been seen previously in ERC tasks and suggest that our task assays similar neurobiological constructs as previously described ERC protocols (Salamone et al., 1991, 1994; Yang et al., 2020). Additionally, we tested the effects of the COMT inhibitor tolcapone and found that it did not significantly modulate ERC performance at any of the doses tested.

To our knowledge, the experiments we describe here are the first report of the effects of acute COMT inhibition alone in an ERC task. Tolcapone has been tested in a PR/concurrent chow ERC task in combination with the vesicular monoamine transporter (VMAT) inhibitor tetrabenazine and it did not modify the decrease in PR responding caused by tetrabenazine (Randall et al., 2014). Our data indicate that COMT activity does not regulate ERC performance to a significant degree in male mice.

It is still possible that COMT may be critically involved in ERC behavior and our experimental conditions were just not optimized to measure the effects of COMT inhibition. For example, we chose to focus on a FR4/concurrent chow ERC task and COMT inhibition may modulate performance in ERC tasks that use different reinforcement schedules or different types of effort (Salamone et al., 1994; Randall et al., 2014; Yohn et al., 2015b). We also used an acute, single-dose treatment strategy to specifically investigate how real-time modulation of COMT activity affects ERC performance. A chronic treatment regimen may uncover behavioral effects due to long-term alterations in COMT function and downstream adaptations. The utilization of genetic models of variable COMT activity may demonstrate a specific role for COMT in ERC performance. Additionally, COMT function is sexually dimorphic in both humans and rodents (Sannino et al., 2017), so COMT may significantly regulate ERC performance in females. Future studies will be designed to address potential sex differences in ERC performance. Moreover, we only used a single strain (C57BL/6J), so our results may also be strain-specific. We chose C57BL/6J mice because they are a commonly used strain for behavioral studies and many of the mutant models we use in our laboratory are on a C57BL/6J background (Carr et al., 2016; Detrait et al., 2016).

Two other possible explanations for the lack of an effect of COMT inhibition on ERC behavior are that the interaction between dopaminergic signaling in the ACC and ERC behavior is: (1) unidirectional; or (2) insignificant. Previous work has indicated that the infusion of a D1 antagonist into the ACC decreases preference for the high cost/high reward option (Schweimer and Hauber, 2006). To our knowledge, selective agonists have not been infused in the ACC, so it is possible that augmenting DA signaling does not alter ERC behavior. In contrast, lesions of the DA innervation within the ACC did not affect ERC behavior (Walton et al., 2005), suggesting that DA modulation may not be a critical factor in ACC processing during effort-related decision making.

We believe the results of acute tolcapone administration reported here are an accurate representation of the effects in this population (young, male C57BL/6J mice) because our validation data indicate we used a robust ERC task. Our current protocol was a modified version of a previously published touchscreen ERC assay (Heath et al., 2015). Also, haloperidol produced the expected decrease in preference for the high-effort high-value option seen in traditional ERC tasks (Salamone et al., 1991) and a recently developed touchscreen version (Yang et al., 2020).

Our data suggest an interesting potential dichotomy in the role of COMT in reward-seeking behavior. While our data show no effect of COMT inhibition on ERC behavior, there are very clear effects of COMT modulation on intertemporal choice and delay discounting in humans (Boettiger et al., 2007; Kayser et al., 2012). To our knowledge, the role of COMT in intertemporal choice has not been tested in animal models. There is evidence that distinct neural circuits regulate delay and effort discounting (Prévost et al., 2010; Klein-Flügge et al., 2016). Additional experimentation will be required to determine the exact role of COMT function in reward-seeking behavior, particularly related to effort and delay discounting.

COMT inhibition has been proposed as a therapeutic strategy for combating cognitive impairment, particularly disorders of cognitive control (Apud and Weinberger, 2007). There is evidence in humans and rodents that COMT inhibition may be beneficial for specific populations (Gasparini et al., 1997; Kayser et al., 2012; McCane et al., 2014) or on specific cognitive tasks (Tunbridge et al., 2004; Byers et al., 2019). Determining the utility of COMT inhibitors will require data showing the effects of these compounds across multiple cognitive domains so that clinical populations that would benefit from this class of compounds can be identified. These experiments represent a potentially important contribution to this literature as the first report on the effects of COMT inhibition on effort-related decision-making.

## Data Availability Statement

The datasets generated by this project are available on request to the corresponding author.

## Ethics Statement

All experiments and procedures were approved by the SoBran, Inc., Fairfax, VA, USA Rangos Animal Care and Use Committee and following the *Guide for the Care and Use of Laboratory Animals*.

## Author Contributions

GC conceived the experiment and supervised the project with JB. AB and AW collected and analyzed the data. AB and GC designed the experiments, interpreted the data, and wrote the manuscript. All authors provided feedback and revised the manuscript.

## Conflict of Interest

JB is an inventor on patents that include novel COMT inhibitors (WO2016123576 and WO2017091818). The remaining authors declare that the research was conducted in the absence of any commercial or financial relationships that could be construed as a potential conflict of interest.

## Abbreviations

ACC: anterior cingulate cortex
COMT: catechol-*O*-methyltransferase
DAT: dopamine transporter
EEfRT: Effort Expenditure for Rewards Task
ERC: effort-related choice
FR: fixed ratio
ip: intraperitoneal
NAc: nucleus accumbens
PFC: prefrontal cortex
PR: progressive ratio
VMAT: vesicular monoamine transporter.
